# UDP-Glucose: A Potential Signaling Molecule in Plants?

**DOI:** 10.3389/fpls.2017.02230

**Published:** 2018-01-09

**Authors:** Henry Christopher Janse van Rensburg, Wim Van den Ende

**Affiliations:** Laboratory of Molecular Plant Biology, KU Leuven Leuven, Belgium

**Keywords:** UDP-glucose, sugar phosphates, sugar signaling, UGPase, UAPase

## Abstract

This perspective paper focuses on the most recent results suggesting a potential role for UDP-Glucose as a signaling molecule in plants. In animals, UDP-Glucose is well-established as an extracellular signaling molecule that is sensed by G-protein coupled receptors, activating several downstream defense mechanisms. Recent studies have shown that abnormal growth occurred in both vegetative and reproductive tissue of plants with reduced UDP-Glucose levels, and this could be rescued by exogenous UDP-Glucose. In plants with increased biomass accumulation, the genes involved in UDP-Glucose production were up-regulated. However, excessive endogenous accumulation of UDP-Glucose induced programmed cell death (PCD), and this could also be obtained by exogenous UDP-Glucose application. Plants with decreased UDP-glucose were insensitive to pathogen induced PCD. We speculate that UDP-Glucose acts as an extracellular signaling molecule in plants, and that it may be perceived as a damage-associated molecular pattern.

## Introduction

Gene expression is regulated by diverse signals and stimuli that are sensed and subsequently transmitted via signaling pathways that ultimately control transcription of genes. For sugar signaling, this starts by sensing the level and nature of a specific sugar. Several mechanisms have been proposed on how these sugars are perceived. Since sugars are readily interconverted in cells, identifying the precise sugar that is sensed is difficult. The mechanisms downstream of sugar sensing is even more complex, and most of the knowledge on sugar signaling in plants were obtained from yeast and mammalian systems.

Carbohydrates like sucrose (Suc) and glucose (Glc) have been linked to their role as signaling molecules during plant development and stress conditions. For Glc, the major energy and carbon source in eukaryotic organisms, this is well-established by the identification of HXK which is the first enzyme in Glc catabolism. This enzyme is now characterized as a true Glc sensor with both signaling and catalytic activities (Jang et al., 1997; Moore et al., 2003). Although no Suc sensor has been identified to date, there seem to be Glc and Suc signaling pathways that function independently of HXK (Chiou and Bush, 1998; Solfanelli et al., 2006). Apart from catabolic products from Suc and carbohydrate reserves, the metabolism of trehalose, specifically trehalose-6-phosphate (T6P), has been suggested as a key regulator of metabolism associated with plant growth and development (Ramon and Rolland, 2007; Lunn et al., 2014). Although a lot of advances have been made, the array of sugar signals and sensors together with their molecular mechanisms that mediate primary signaling is yet to be fully explored.

In animals, UDP-Glucose (UDP-Glc) acts as extracellular signaling molecule via G-protein-coupled receptors (Chambers et al., 2000; Freeman et al., 2001), but little has been reported in plants. Recently several studies in plants suggest a potential role for UDP-Glc in biomass accumulation, growth and development, and programmed cell death (PCD) (Chivasa et al., 2013; Wang et al., 2015; Wai et al., 2017; Xiao et al., 2017). Although changes in UDP-Glc levels were associated with these responses, little is known on how exactly, and if this can be directly associated with UDP-Glc. Here, we consider the recent advances on UDP-Glc as a potential signaling molecule in plants and identify the gaps in our knowledge.

## The Search for Sucrose Receptors Continues

Sucrose was proposed as a signaling molecule in plants (Pontis, 1978; Koch, 2004; Wind et al., 2010) but it is rapidly metabolized by invertases and sucrose synthases (SuSys) (Horacio and Martinez-Noel, 2013), and therefore its breakdown products Glc, UDP-Glc and fructose (Fru) may potentially act as signaling molecules as well (Hummel et al., 2009). Suc also serves as a substrate for polysaccharide synthesis, making it extremely difficult to distinguish between its role in serving as a building block for storage and polysaccharide synthesis and/or its involvement as a signaling molecule. Above a certain Suc threshold, *de novo* synthesis of numerous genes and proteins occurs, providing evidence of its regulatory role (Pollock et al., 2003).

The role of Suc as a signaling molecule became clearer in experiments where exogenous application of Suc, but not a (combination of) hexoses, led to significant responses. Increased levels of Suc are known to induce the expression of genes involved in starch biosynthesis such as the ADP-Glc pyrophosphorylase (AGPase) in several species, however, from these reports it is not clear whether this response was specific to Suc (Harn et al., 2000; Wang et al., 2001; Nagata et al., 2012). It is also proposed that Suc controls its own synthesis indirectly, as Suc applied to excised leaves upregulated the UDP-Glc pyrophosphorylase (UGPase), producing the substrate for sucrose phosphate synthase (SPS) (Ciereszko et al., 2001). Suc has also been linked to the positive regulation of nitrate and ammonium transport in Arabidopsis, however, hexose sugars were able to produce similar results (Lejay et al., 2008).

Even though, Suc is the main sugar transported from source to sink tissue in plants, several sugar responses depend on Glc and other signaling sugars directly, or indirectly through energy and metabolite sensors, through the activity of invertases and SuSys (Cho et al., 2009; Ruan, 2014). Moreover, multiple signaling pathways for Suc may exist (Tognetti et al., 2013; Lastdrager et al., 2014). Invertases and SuSys are the only known enzymes for Suc cleavage, producing Glc + Fru and UDP-Glc and Fru, respectively. Their potential signaling roles may contribute to an even more complex network, relying on a combination or a certain balance of these sugars. The fact that no Suc receptor has been identified so far might suggest that it is in fact not Suc but the breakdown products UDP-Glc and/or Fru that act as signals. There is no evidence suggesting that fructokinases are involved in sugar signaling, however, it has been proposed that a nuclear localized fructose 1-6-bisphosphatase (FBP/FIS1, FRUCTOSE-INSENSITIVE1) acts as Fru sensor in Arabidopsis (Cho and Yoo, 2011).

## Udp-Glc Levels Affect Growth and Development, and the Response to Stress Conditions in Plants

**FIGURE 1:**
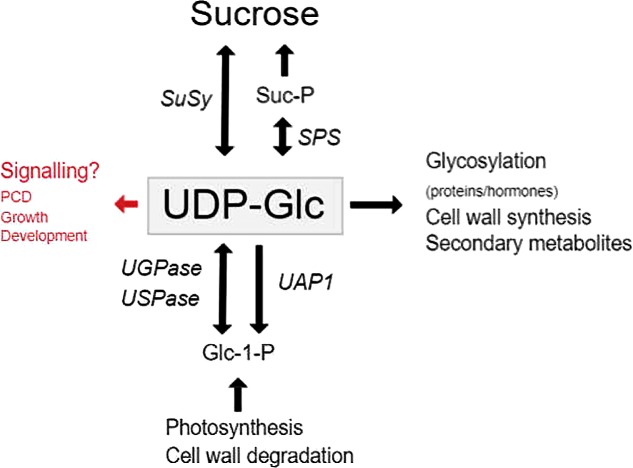
The functions and synthesis of UDP-Glc in plants. The major roles of UDP-Glc in plant cells, and the genes involved in UDP-Glc metabolism. Genes are indicated by italics.

Uridine 5′ -diphosphate-glucose (UDP-Glc) serves as the key substrate in the synthesis of both Suc and polysaccharides, and serves as the Glc donor for many glycosylation reactions (**Figure 1**). In animals, UDP-Glc serves as an extracellular signaling molecule that activates several pathways (Harden et al., 2010), however, in plants very little has been reported. The majority of UDP-Glc formed in plants are due to three distinct classes of enzymes namely UDP-Glc pyrophosphorylase (UGPase or UGP in short), UDP-sugar pyrophosphorylase (USPase or USP in short) and SuSy (**Figure 1**). UDP-Glc is mainly synthesized from UTP and Glucose-1-phosphate (G1P) through UGPase in source tissues and formed together with Fru via the degradation of Suc by SuSy in sink tissues.

The expression of UGPase, USPase and SuSy are subject to fine regulation in all plants studied thus far (Koch, 2004; Geisler-Lee et al., 2006; Litterer et al., 2006; Meng et al., 2007). These genes are known to be differentially expressed under stress conditions (Ciereszko et al., 2001; Ciereszko and Kleczkowski, 2002; Baud et al., 2004; Chang et al., 2005; Meng et al., 2007). In a recent study, it was demonstrated that increased biomass accumulation in sugarcane was associated with the rapid conversion of Suc to UDP-Glc, serving as building blocks for cell wall synthesis (Wai et al., 2017). The genes involved in UDP-Glc and Suc metabolism were differentially expressed between the high biomass and low biomass variety. Those involved in the conversion of Glucose-6-phosphate (G6P) to UDP-Glc and several SuSys were up-regulated in the high biomass cultivar (Wai et al., 2017), indicating a clear association between extra UDP-Glc synthesis and increased biomass. In Arabidopsis with mutant alleles for both *AtUGP1* and *AtUGP2*, UDP-Glc content was reduced to 48% compared to WT, and showed extreme reduction in vegetative growth and male sterility even though SuSy and USPase activities remained unchanged (Park et al., 2010). Exogenous Suc was able to restore vegetative growth, probably through the breakdown of Suc by SuSy, but not male fertility, whereas UDP-Glc could also restore male fertility. Similarly, UDP-Glc and UDP-galactose were able to reverse the inhibition of pollen germination in UGPase/USPase inhibitor studies (Decker et al., 2017). This suggests that UGPase and UDP-Glc act as important regulators of plant development and growth.

It has previously been shown that a UGPase from Arabidopsis acts as a novel PCD regulator (Chivasa et al., 2013). It was suggested that the Suc-induced *UGP1* is critical in the regulation of PCD in Arabidopsis during pathogen infection, and *UGP1* mutants are insensitive to pathogen induced PCD (Chivasa et al., 2013). In a more recent study however, a rice UDP-*N*-acetylglucosamine pyrophosphorylase 1 (*UAP1*) mutation (*spl29*) caused lesion mimics and subsequent leaf senescence (Wang et al., 2015). UAP1 catalyzes the reversible reaction of GlcNac1P and GalNAc1P to their respective UDP-sugars (Yang et al., 2010), and also converts UDP-Glc to UTP and G1P *in vivo* and *in vitro* (Xiao et al., 2017). The *spl29* mutant showed abnormal agronomical traits and developed lesions in conjunction with accumulative levels of ROS typical of PCD (Xiao et al., 2017). This was attributed to the inability to degrade UDP-Glc, leading to excessive build-up of UDP-Glc in these plants. Accumulative UDP-Glc is believed to have triggered PCD via caspase-like activities and ER stress. Exogenously applied UDP-Glc and UDP-GlcNAc induced PCD and ROS accumulation even further in both WT and *spl29* mutants. The correlation of these results suggests that it might in fact be the subsequent accumulation of UDP-Glc that regulates pathogen induced PCD in Arabidopsis instead of the proposed activity of UGP1. Overall, PCD formation can also be attributed to ROS accumulation and subsequent ROS signaling. Upon pathogen infection, WT plants showed excessive reprogramming of proteins involved in chloroplastic photosynthesis, whereas in *UGP1* mutants these proteins were not affected, pointing toward ROS formation within the chloroplast during PCD (Chivasa et al., 2013). The ROS accumulation in the chloroplast can be attributed to metabolic imbalances caused by altered photosynthetic gene expression.

No plant plasma membrane (PM) UDP-Glc transporter has been identified to date, although UDP-Glc transporters exist in the endoplasmic reticulum membrane (Reyes et al., 2010). However, we are not aware of any ongoing research into PM UDP-Glc transporters. In any case, judging on the overall size of the molecule and the presence of charged phosphates, it can be expected that UDP-Glc cannot simply cross the PM by simple diffusion. Anderson and Ray (1977) and Mueller and Maclachlan (1982) demonstrated that an exogenous supply of radiolabeled UDP-Glc was not taken up by intact cells, and only damaged cells were able to utilize the available extracellular UDP-Glc. However, this does not exclude the presence of dedicated PM UDP-Glc transporters in other cell types than those investigated by Anderson and Ray (1977) and Mueller and Maclachlan (1982). Deeper investigations into PM UDP-Glc transporters are warranted. It will also be interesting to follow up on what exactly happens with exogenously supplied UDP-Glc once it enters the apoplastic continuum (apoplastic fluid analyses as a function of time). A metabolic conversion by apoplastic UDP-Glc metabolizing enzymes (e.g., apoplastic SuSy, among others) cannot be excluded.

In maize, an alternative function for SuSy, apart from its catalytic activity, has been suggested (Subbaiah et al., 2006). Mitochondrial SuSys (*SH1* and *SUS1*) interacting with the anion channel on the mitochondrial membrane in an anoxia-enhanced manner, are believed to be involved in the regulation of solute fluxes in or out of the mitochondria (Subbaiah et al., 2006). Interestingly, both SuSy and the anion channel were also present in the nucleus (Subbaiah et al., 2006), suggesting a potential role for SuSy and/or UDP-Glc, via anoxia-dependent signaling, in the PCD pathway. This can potentially explain the accumulation of ROS and subsequent PCD formation in plants with increased UDP-Glc levels.

In this instance it would be interesting to confirm whether exogenously applied UDP-Glc can in fact be taken up by plant cells. The fact that exogenous application restored the phenotype of these plants suggests that UDP-Glc is either taken up into the cells or sensed extracellularly to restore the growth defects. Following the changes in gene expression and metabolite levels after exogenous UDP-Glc application will also shed light on the potential activation of signaling pathways that translate this signal to plant metabolism and physiological adaptation. The effect of exogenous UDP-Glc application on the growth of WT Arabidopsis can also provide insight on whether it acts as a signal or merely a metabolic building block.

## Udp-Glc: Another Promiscuous Substrate for Uap?

UDP-GlcNac, produced by UAP, is the precursor for glycoprotein and glycolipid synthesis. In Arabidopsis, UAP1 can reversibly convert GlcNAc1P and GalNAc1P to UDP-GlcNAc and UDP-GalNAc, whereas UAP2 converts GlcNAc1P, GalNAc1P and to some extent G1P into their corresponding UDP-sugars (Yang et al., 2010; Decker and Kleczkowski, 2017). UAP2 showed higher affinity for UDP-Glc than for G1P, suggesting a role in metabolizing UDP-Glc rather than producing it Decker and Kleczkowski (2017). In rice, UAP1 was initially shown to reversibly convert GlcNAc1P to UDP-GlcNAc and UDP-GalNAc to GalNAc1P, respectively (Wang et al., 2015). The forward reaction for GalNAc1P was not tested due to lack of availability of substrate at that time. More recently, it was shown to breakdown UDP-Glc to G1P, leading to an excessive UDP-Glc accumulation in the mutant (Xiao et al., 2017). Phylogenetic analysis revealed that the rice UAP1 had high identity to its homolog UAP2 (88%) and to the Arabidopsis UAP1 and UAP2 (both 78%) (Wang et al., 2015). The ability of the rice UAP1 to use UDP-Glc as substrate was particularly interesting (Xiao et al., 2017), as the Arabidopsis UAP2 showed very low activity with G1P as substrate with high K_m_ (3.2 mM), but showed a low K_m_ (0.21 mM) for UDP-Glc (Decker and Kleczkowski, 2017). Arabidopsis UAP2 also shows more common amino acids for UDP binding than for sugar binding. This suggests that the rice UAP1 and Arabidopsis UAP2 can be considered functional homologs, being more favorable for the breakdown of UDP-Glc, similar to UGPase.

Clearly UAP can catalyze the conversion of a broad range of substrates. The ability of some UAP enzymes to convert UDP-Glc to G1P (Xiao et al., 2017) remains somewhat mysterious and requires further investigation to determine whether this indeed widely occurs in plants, and deeper research is needed to the actual reason for this reaction. Also, the excessive accumulation of UDP-Glc in the *UAP1* mutant lines needs further investigation, as a compensation by UGPase, USPase, or SuSy activities would be expected, but apparently does not seem to occur. The UDP-Glc accumulation in these mutants might also be indirectly caused by the lowered levels of UDP-GlcNAc/UDP-GalNAc required for protein glycosylation (e.g., UGPase/USPase/SuSy). It will be interesting to investigate whether Arabidopsis *UAP2* mutants show similar UDP-Glc accumulation, since UAP2 shows a high affinity for UDP-Glc *in vivo* (Decker and Kleczkowski, 2017). Further, the expression levels of *UGPase*, *USPase*, and *SuSy* together with their enzyme activity should be followed in rice *UAP1* (*spl29*) and Arabidopsis *UAP2* mutants as compared to WT, to pinpoint the reason for UDP-Glc accumulation.

## Udp-Glc Signaling, Or Merely Metabolic Imbalance?

Most studies analyzing genes contributing to the UDP-Glc pool do not consider changes in metabolites other than UDP-Glc (Chivasa et al., 2013; Wang et al., 2015; Xiao et al., 2017). This poses the question as to whether UDP-Glc is directly involved in signaling or is it merely the disturbance in sugar levels that fulfills the signaling role? In the *UAP1* mutant (Xiao et al., 2017), the inability to degrade UDP-Glc to G1P in seedlings can deplete the levels of G1P, also known to be an inhibitor, in combination with T6P, of SnRK1 (Nunes et al., 2013). Although somewhat controversial, accumulating UDP-Glc can favor Suc synthesis through SuSys reversible action, leading to increased Suc which is also a proposed activator of SnRK1 (Purcell et al., 1998; Rolland et al., 2006). Interestingly, SnRK1 has recently been linked to a potential role in the activation of autophagy via the target of rapamycin (TOR) signaling pathway by inhibiting the activity of TOR in Arabidopsis (Soto-Burgos and Bassham, 2017). TOR is a negative regulator of autophagy under normal conditions. Alterations in G1P/Suc levels may thus also contribute to autophagy via SnRK1 and TOR, causing PCD in UDP-Glc accumulating plants. It will be interesting in this regard to determine what effect high concentrations of endogenous UDP-Glc has on T6P, G1P and Suc, particularly in the UAP1 and UGPase mutants. Comparing this data set with one from exogenously applied UDP-Glc will also help in understanding the mechanisms involved in UDP-Glc induced PCD. By studying the metabolic profiles together with SnRK1 and TOR activity in these mutants can provide valuable information on whether UDP-Glc is directly perceived as a signal or act only as intermediate in this pathway.

In conclusion, accumulation of UDP-Glc can cause an imbalance in other metabolites that are known to be involved in sugar signaling, instead of being a signaling molecule itself. Both these hypothetical scenarios provide alternative signaling pathways that can arise from increased levels of UDP-Glc.

## Evidence for Potential Udp-Sugar Receptors in Plants?

UDP-sugars, or the so called activated sugars, are high energy donor substrates for several biosynthetic reactions in cells. These sugars also play an active role in the glycosylation of proteins in the secretory pathway of the endoplasmic reticulum. In animals, it is well-established that these sugars interact with receptors on the cell surface, and several nucleotide-activated cell surface receptors have been identified with a wide variety of downstream responses (Ralevic and Burnstock, 1998; Burnstock, 2007; Harden et al., 2010). In plants, however, no potential receptor has been identified for these sugars yet.

In animals, the UDP receptor, P2Y_14_, shows a high affinity for extracellular UDP-Glc compared to other nucleotides and nucleotide sugars (Chambers et al., 2000). This receptor has been associated with several downstream cell signaling responses in animals (Krzemiński et al., 2008; Fricks et al., 2009; Harden et al., 2010). The most interesting response is the activation of MAP kinase signaling in the presence of UDP-Glc (Fricks et al., 2009). MAP kinases are known to be involved in several biotic and abiotic stress response mechanisms in plants (Kovtun et al., 2000; Liu et al., 2000; Zhang et al., 2012). Interestingly, a clear correlation between MAP kinase cascades and ROS signaling has been found in plants (Pitzschke and Hirt, 2006). By manipulating the MAP kinase cascades, ROS signaling was induced, and ROS accumulation on the other hand activated MAP kinases. Both these processes are also involved in the activation of PCD in plants. This may explain the ROS accumulation in plants with high UDP-Glc levels, if a UDP-Glc receptor would exist in plants. There is no information on extracellular UDP-Glc in plants. Can the UDP-Glc released by dead cells potentially be perceived as a damage-associated molecular pattern (DAMP), similar to what is proposed for fructans (Versluys et al., 2017)? This might also explain the above-mentioned responses to exogenous application of UDP-Glc in plants.

In animals, UDP-Glc receptors seem to play a crucial role in response to stress conditions by activating several downstream cascades serving as protective mechanisms. In plants, however, it is still unclear as to whether UDP-Glc plays any role as a signal molecule, and no receptor has been associated with this nucleotide sugar either. In Arabidopsis, G-protein-receptors have been associated with signal transduction of external sugars and subsequent autophagy (Jeffrey et al., 2008; Tunc-Ozdemir and Jones, 2017; Yan et al., 2017), providing potential candidates similar to those of animals.

## Conclusion

Fluctuations in sugar levels are clearly associated with metabolic responses, however, the exact mechanisms of how these sugar levels are perceived are not yet fully understood. Glc, Suc, and T6P are the most studied sugars for their role in metabolic signaling in plants, however, several other sugars and intermediates have been proposed as signaling candidates. UDP-Glc has been proposed as potential intracellular mediator of ROS signaling and PCD. Alterations in endogenous levels of UDP-Glc had several downstream responses which could also be simulated with exogenous UDP-Glc. This points to a potential signaling role of UDP-Glc in plants. In animals, UDP-Glc functions as an extracellular signaling molecule perceived by receptors that triggers several downstream kinases. These findings suggest that UDP-Glc can play a similar role in plants to what is found in animals. This opens the door for future work on UDP-Glc as potential signaling molecule and unraveling the mode of perception of this sugar nucleotide in plants.

## Author Contributions

All authors listed have made a substantial, direct and intellectual contribution to the work, and approved it for publication.

## Conflict of Interest Statement

The authors declare that the research was conducted in the absence of any commercial or financial relationships that could be construed as a potential conflict of interest.
